# A Case of Torticollis in an 8-Month-Old Infant Caused by Posterior Fossa Arachnoid Cyst: An Important Entity for Differential Diagnosis

**DOI:** 10.3390/pediatric13020027

**Published:** 2021-04-12

**Authors:** John K. Yue, Taemin Oh, Kasey J. Han, Diana Chang, Peter P. Sun

**Affiliations:** 1 Department of Neurosurgery, University of California San Francisco, San Francisco, CA 94122, USA; taemin.oh@ucsf.edu (T.O.); kasey.han@ucsf.edu (K.J.H.); diana.chang@ucsf.edu (D.C.); peter.sun@ucsf.edu (P.P.S.); 2 Division of Pediatric Neurosurgery, Department of Neurosurgery, University of California San Francisco, San Francisco, CA 94122, USA

**Keywords:** arachnoid cyst, differential diagnosis, posterior fossa, torticollis

## Abstract

Torticollis is a clinical diagnosis with heterogeneous causes. We present an unusual case of acquired torticollis in an 8-month-old female infant with a large cerebellopontine angle arachnoid cyst. Symptoms resolved after surgical fenestration. Non-traumatic acquired or new-onset torticollis requires brain imaging, and posterior fossa lesions are an important entity in the differential for pediatric clinicians.

## 1. Introduction

Torticollis is a clinical diagnosis defined by unnatural, asymmetric head and neck position or tilt, also known as head/neck dystonia, with an estimated incidence of 1.3% in childhood [1]. Oftentimes, the neck musculature is contracted, and the chin is tilted toward the opposite direction. The underlying cause may be congenital or acquired, and encompasses a wide differential that includes multiple systems: trauma, infection, inflammatory, vascular, osseous, ligamentous, muscular, ocular, neurological, psychiatric or due to drug reaction [2]. It is important to distinguish torticollis originating from the central nervous system from those in other systems. These lesions can cause focal dystonias and may originate from the basal ganglia, brainstem, cerebellum or cervical spine [2]. Treatment involves addressing the underlying condition, and can be curative.

Congenital torticollis presents at or soon after birth, and is defined as any postural deformity of the neck that develops prenatally, e.g., craniocervical bony/vertebral abnormalities or due intrauterine malpositioning or birth trauma that causes congenital muscle damage (commonly to the sternocleidomastoid muscle) [3]. In contrast, acquired torticollis appears during childhood and is secondary to a progressive disorder or mass lesion, e.g., trauma, infection, tumors, neurogenic, ocular and other disorders [4]. One location for such lesions is the posterior fossa, which contains the cerebellum and brainstem. Tumors of the posterior fossa account for 50–55% of pediatric intracranial tumors [5]. As these lesions rarely regress without intervention, the patient’s torticollis may continue to worsen over time, and deficits may become permanent. Early diagnosis of the underlying condition is thus of the utmost importance to ensure proper treatment and recovery.

Here, we report a rare case of a large cerebellopontine angle (CPA) arachnoid cyst causing acquired torticollis in an 8-month-old male infant, who showed a significant improvement in symptoms after operative fenestration and resolution by 2-week follow-up. We discuss the considerations for the vigilant pediatric clinician for the timely diagnosis and management of this rare entity amongst the differential for torticollis.

## 2. Case Presentation

### 2.1. History and Physical Exam

An 8-month-old male infant presented to the emergency department with 2 months of increased left neck muscle tone and rightward chin tilt consistent with acquired torticollis. Symptomatically, the patient had chronic cough, congestion, drooling, disordered breathing and poor weight gain for the same amount of time. In terms of birth history, the patient was born full term at 40 weeks with an uncomplicated pregnancy with proper prenatal care, planned cesarean section, and standard neonatal discharge to home.

On examination, the patient had normal vital signs but was notably fussy with a hoarse cry, drooling, increased leftward neck flexion and rotation and rightward chin tilt, which raised the concern for neurologic deficits. Basic labs including complete blood count, basic metabolic panel and coagulation studies were within normal limits.

### 2.2. Preoperative Imaging

**Figure 1:**
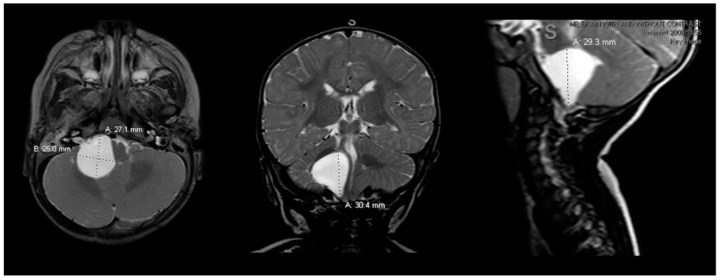
Preoperative brain MRI scan. The preoperative brain MRI scan (left = axial; middle = coronal; right = sagittal) T2 sequences showed a large 2.5 × 2.7 × 3.0 cm homogeneous, hyperintense right cerebellopontine angle cystic lesion causing mass effect on the right pons and medulla. T1 sequences (not shown) were hypointense for the cyst, without contrast enhancement on gadolinium scans. MRI = magnetic resonance imaging.

Preoperative magnetic resonance imaging (MRI) of the brain and cervical spine was obtained as the patient’s respiratory difficulties and failure to thrive suggested a lesion localizing to the brainstem. MRI showed a large cystic lesion at the right CPA measuring 2.7 × 2.5 × 3.0 cm (anteroposterior (AP) × transverse (TV) × cephalocaudal (CC), respectively), hypointense on T1 and hyperintense on T2, without gadolinium enhancement, diffusion restriction or susceptibility artifact, most consistent with an arachnoid cyst (Figure 1). The lesion was causing overt flattening of the pons and mass effect along the length of the right superoanterior and lateral medulla, mild mass effect on the right cerebellum, and displacement and mild effacement of the fourth ventricle. There was no abnormal vascularity, syrinx or chiari malformation or extension down to the cervical spine. Ventricles were otherwise normal in morphology and size.

### 2.3. Operative Fenestration

The patient was admitted to the pediatric intensive care unit for neurologic monitoring and underwent a right retrosigmoid craniotomy for fenestration of the arachnoid cyst. Upon opening of the dura, cerebrospinal fluid (CSF) was allowed to drain for brain relaxation. Dynamic retraction revealed the cyst walls, with cranial nerve 7 (facial nerve), cranial nerve 8 (vestibulocochlear nerve), as well as the lower cranial nerve complexes embedded within the cyst and posterior cyst wall. The cerebellum was protected, and the posterolateral cyst wall was sharply opened with egress of CSF. The inner walls of the cyst were lined with arachnoid and did not contain any abnormal tissue. CSF cytology was sent which showed no evidence of malignancy. The cyst was fenestrated toward the prepontine cistern and thoroughly irrigated prior to dural closure and bone replacement. There were no changes in neuromonitoring.

### 2.4. Postoperative Management and Outcome

**Figure 2:**
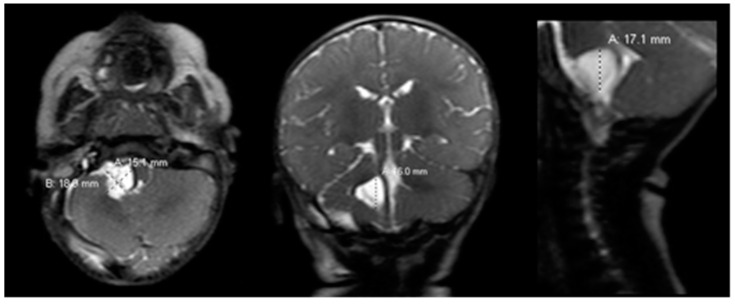
Postoperative brain MRI scan. The postoperative brain MRI scan on postoperative day 1 (left = axial; middle = coronal; right = sagittal) T2 sequences showed interval decompression of the prior known arachnoid cyst with decreased mass effect. MRI = magnetic resonance imaging.

Postoperatively, the patient returned to the pediatric intensive care unit and was extubated. Postoperative rapid-sequence MRI showed a well-decompressed cyst with decreased mass effect on the pons, medulla and cerebellum (Figure 2).

**Figure 3:**
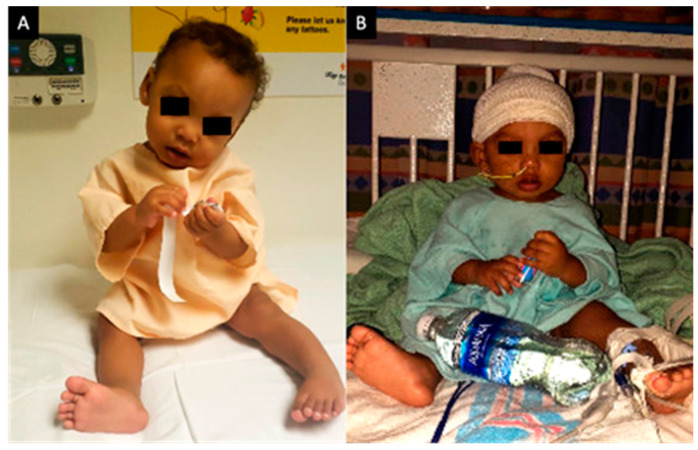
An 8-month-old male infant with torticollis. Panel (**A**) shows the preoperative patient with asymmetric leftward neck flexion and rotation and rightward chin tilt. Panel (**B**) shows the postoperative patient on POD 1 with resolution of prior head/neck flexion and chin tilt, as the neck is now midline. POD = postoperative day.

The patient’s neck posture and head tilt improved significantly on postoperative day (POD) 1 (Figure 3B) compared to upon emergency department admission (Figure 3A), and continued to improve with daily speech and language therapy, physical therapy and occupational therapy. He was co-managed with the pediatric inpatient team. Initially per speech therapy recommendations the patient was managed with nothing by mouth (nil per os) and nasogastric feeding tube due to continued drooling and coughing during bottle feeds and concern for subclinical aspiration. Importantly, the patient was continued on daily nipple stimulation in order to maintain appropriate oral phase response, and his drooling progressively improved. A video fluoroscopic swallow study on POD 5 found mild pharyngeal phase dysphagia only when fatigued. The patient was cleared for nipple/bottle feeding and age-appropriate puree and foods, with strict maintenance of upright posture when feeding, and a rest break after every ounce of nippling. The patient was discharged home in good condition on POD 6.

At his 2-week follow-up evaluation, the patient’s head and chin were straight, their cry was no longer hoarse, and there was no abnormal drooling. He was tolerating age-appropriate oral feeds. On physical therapy evaluation, he could rotate his head symmetrically in both directions and was meeting his neonatal motor milestones. There was some expected mild muscle weakness of the right lateral neck flexors compared to the left, which is expected to improve with continued physical and occupational therapy.

### 2.5. Informed Consent

The patient’s legally authorized decision makers (parents) provided written informed consent for inclusion of the patient’s case in research and publication, which is held by the authors/investigators in the patient’s medical record.

## 3. Discussion

Arachnoid cysts are congenital lesions arising during development from splitting of the arachnoid membrane, and do not communicate with the ventricles or subarachnoid space. The incidence is 0.5–2% in the general population, and 1% of all intracranial masses. Arachnoid cysts are commonly diagnosed in childhood, incidentally by imaging, and the majority are asymptomatic and do not require operative intervention [6]. By location, most lie in the sylvian fissure (49%) and CPA (11%), followed by supracollicular (9%), vermian (9%), sellar/suprasellar (9%), interhemispheric (5%), cerebral convexity (4%), and clival (3%) [7]. Evaluation by MRI is preferred to computed tomography (CT), as MRI can differentiate between CSF versus neoplastic cyst fluid, and is preferred in children due to the absence of ionizing radiation. Cysts may enlarge, rupture or hemorrhage over time, resulting in symptoms requiring urgent evaluation. For incidental arachnoid cysts, however, serial MRIs and clinical follow-up are recommended to evaluate for growth and symptomatology, respectively. Treatment is only recommended for enlarging or symptomatic cysts due to associated procedural risks, including (1) microsurgical or (2) endoscopic fenestration into basal cisterns, or (3) shunting to peritoneum. While still a matter of debate and surgeon preference, reports have shown that cyst fenestration by microsurgical or endoscopic methods may reduce the risk of requiring a shunt in children [8,9]. Furthermore, due to the anatomy of the CPA, craniotomy with microsurgical fenestration has been considered definitive treatment [10].

Importantly, any mass lesion arising in the posterior fossa can produce signs and symptoms related to the compression of nearby neurovascular structures or by obstruction of CSF flow. These symptoms can include non-specific signs, such as vomiting, macrocephaly, ataxia and torticollis, or more localizing deficits, such as ocular palsy, cranial nerve deficits and corticospinal or corticobulbar symptoms. Torticollis can occur as a compensatory mechanism for diplopia, hearing loss or due to direct compression on the brainstem or cerebellar neural elements including the accessory nerve (cranial nerve 11). In our case, the patient’s symptoms of fussiness, drooling and inability to maintain control of the head and neck may have been due to corticobulbar and corticospinal tract dysfunction. Notably, the descending corticobulbar tracts innervate cranial nerves 9, 10, 11 and 12, which include swallowing and the maintenance of secretions, which were symptomatic in our patient. Additionally, while the patients’ face was symmetric on exam, the traversing cranial nerve 7 and 8 complexes seen in the cyst would have likely resulted in facial motor and hearing deficits (hearing was not assessed preoperatively), which could have exacerbated the torticollis.

To date, we have found only five definitive reports of posterior fossa arachnoid cysts causing torticollis that underwent surgical treatment [11,12]. Of these, only three described patients below the age of 2 years. Fulkerson et al. report a case of an 8-month-old male with occipital plagiocephaly and torticollis with a large midline arachnoid cyst extending into the middle cranial fossa who underwent cysto-ventricular shunt [13]. Per et al. describe a 16-month-old male with macrocephaly and 6 months of torticollis with a large right posterior fossa arachnoid cyst extending to the dorsal cervical 3–4 spinal level, who underwent cystoperitoneal shunt with significant postoperative improvement in symptoms [2]. Hanrahan et al. describe a 2-month-old male with increasing head circumference, difficulty feeding, gastroesophageal reflux and a large right posterior fossa arachnoid cyst and obstructive hydrocephalus who initially underwent endoscopic third ventriculostomy, followed by cyst fenestration for torticollis, with resolution of symptoms [11].

Our patient represents an unusual case of torticollis secondary to a posterior fossa arachnoid cyst, which was successfully managed through open cyst fenestration. While the case of posterior fossa arachnoid cyst presenting with torticollis is rare, our case report adds to the literature to show that arachnoid cysts should be included in the differential for acquired and progressive torticollis. Imaging is facile, especially with the advent of rapid brain MRIs which can be acquired in under 5–10 min. For the vigilant clinician, recognition of the signs and symptoms of torticollis, with prompt imaging and neurosurgical referral if posterior fossa arachnoid cyst is found, can lead to good outcomes with surgical intervention, including full symptomatic resolution. It is also important to note from our report that even after surgery and symptomatic improvement, continued physical therapy, occupational therapy, and speech therapy, and referral to outpatient follow-up with neurosurgery, neurology, audiology and multidisciplinary services, as well as periodic brain imaging, will be of high importance for continued treatment and surveillance.

Of note, ultrasound imaging is often utilized for the evaluation of muscular torticollis, which may elucidate causes for primary and secondary torticollis [14,15]. Ultrasound evaluation was not available in the emergency room at the time of our patient’s presentation, which was a limitation to the diagnostic workup of torticollis.

## 4. Conclusions

Posterior fossa arachnoid cyst is a rare but important differential diagnosis for acquired torticollis. For the vigilant pediatric clinician, non-traumatic, acquired and/or new-onset torticollis requires prompt brain imaging, with urgent referral to neurosurgery if arachnoid cyst is present. Surgical outcomes are generally excellent for the symptomatic control and/or resolution of acquired torticollis. Patients should be referred to outpatient follow-up with rehabilitation, speech and appropriate multidisciplinary services for continued evaluation and surveillance.

## Data Availability

Not applicable.
